# Marginal Likelihoods in Phylogenetics: A Review of Methods and Applications

**DOI:** 10.1093/sysbio/syz003

**Published:** 2019-01-22

**Authors:** Jamie R Oaks, Kerry A Cobb, Vladimir N Minin, Adam D Leaché

**Affiliations:** 1 Department of Biological Sciences and Museum of Natural History, Auburn University, Auburn, AL 36849, USA; 2 Department of Statistics, University of California, Irvine, CA 92697, USA; 3 Department of Biology and Burke Museum of Natural History and Culture, University of Washington, Seattle, WA 98195, USA

**Keywords:** Marginal likelihood, model choice, phylogenetics

## Abstract

By providing a framework of accounting for the shared ancestry inherent to all life, phylogenetics is becoming the statistical foundation of biology. The importance of model choice continues to grow as phylogenetic models continue to increase in complexity to better capture micro- and macroevolutionary processes. In a Bayesian framework, the marginal likelihood is how data update our prior beliefs about models, which gives us an intuitive measure of comparing model fit that is grounded in probability theory. Given the rapid increase in the number and complexity of phylogenetic models, methods for approximating marginal likelihoods are increasingly important. Here, we try to provide an intuitive description of marginal likelihoods and why they are important in Bayesian model testing. We also categorize and review methods for estimating marginal likelihoods of phylogenetic models, highlighting several recent methods that provide well-behaved estimates. Furthermore, we review some empirical studies that demonstrate how marginal likelihoods can be used to learn about models of evolution from biological data. We discuss promising alternatives that can complement marginal likelihoods for Bayesian model choice, including posterior-predictive methods. Using simulations, we find one alternative method based on approximate-Bayesian computation to be biased. We conclude by discussing the challenges of Bayesian model choice and future directions that promise to improve the approximation of marginal likelihoods and Bayesian phylogenetics as a whole.

Phylogenetics is rapidly progressing as the statistical foundation of comparative biology, providing a framework that accounts for the shared ancestry inherent in biological data. Soon after phylogenetics became feasible as a likelihood-based statistical endeavor (Felsenstein 1981), models became richer to better capture processes of biological diversification and character change. This increasing trend in model complexity made Bayesian approaches appealing, because they can approximate posterior distributions of rich models by leveraging prior information and hierarchical models, where researchers can take into account uncertainty at all levels in the hierarchy.

From the earliest days of Bayesian phylogenetics (Rannala and Yang 1996; Mau and Newton 1997), the numerical tool of choice for approximating the posterior distribution was Markov chain Monte Carlo (MCMC). The popularity of MCMC was due, in no small part, to avoiding the calculation of the marginal likelihood of the model—the probability of the data under the model, averaged, with respect to the prior, over the whole parameter space. This marginalized measure of model fit is not easy to compute due to the large number of parameters in phylogenetic models (including the tree itself) over which the likelihood needs to be summed or integrated.

Nonetheless, marginal likelihoods are central to model comparison in a Bayesian framework. Learning about evolutionary patterns and processes via Bayesian comparison of phylogenetic models requires the calculation of marginal likelihoods. As the diversity and richness of phylogenetic models has increased, there has been a renewed appreciation of the importance of such Bayesian model comparison. As a result, there has been substantial work over the last decade to develop methods for estimating marginal likelihoods of phylogenetic models.

The goals of this review are to (1) try to provide some intuition about what marginal likelihoods are and why they can be useful, (2) review the various methods available for approximating marginal likelihoods of phylogenetic models, (3) review some of the ways marginal likelihoods have been applied to learn about evolutionary history and processes, (4) highlight some alternatives to marginal likelihoods for Bayesian model comparison, (5) discuss some of the challenges of Bayesian model choice, and (6) highlight some promising avenues for advancing the field of Bayesian phylogenetics.

## What Are Marginal Likelihoods and Why Are They Useful?

A marginal likelihood is the average fit of a model to a data set. More specifically, it is an average over the entire parameter space of the likelihood weighted by the prior. For a phylogenetic model ${M_{}}$ with parameters that include the discrete topology (${T_{}}$) and continuous branch lengths and other parameters that govern the evolution of the characters along the tree (together represented by ${\theta_{}}$), the marginal likelihood can be represented as

(1) \begin{align*} p({D} {\,|\,} {M_{}}) = \sum\limits_{{T_{}}} \int_{{\theta_{}}} p( {D} {\,|\,} {T_{}}, {\theta_{}}, {M_{}}) p({T_{}}, {\theta_{}} {\,|\,} {M_{}}) {\mathrm{d}{{\theta_{}}}}, \label{eq:marginalLikelihood} \end{align*}

where ${D}$ are the data. Each parameter of the model adds a dimension to the model, over which the likelihood must be averaged. The marginal likelihood is also the normalizing constant in the denominator of Bayes’ rule that ensures the posterior is a proper probability density that sums and integrates to one:

(2) \begin{align*} p({T_{}}, {\theta_{}} {\,|\,} {D}, {M_{}}) = \frac{ p({D} {\,|\,} {T_{}}, {\theta_{}}, {M_{}}) p({T_{}}, {\theta_{}} {\,|\,} {M_{}}) }{ p({D} {\,|\,} {M_{}}) }. \label{eq:bayesRule} \end{align*}

Marginal likelihoods are the currency of model comparison in a Bayesian framework. This differs from the frequentist approach to model choice, which is based on comparing the maximum probability or density of the data under two models either using a likelihood ratio test or some information-theoretic criterion. Because adding a parameter (dimension) to a model will always ensure a maximum likelihood at least as large as without the parameter, some penalty must be imposed when parameters are added. How large this penalty should be is not easy to define, which has led to many different possible criteria, e.g., the Akaike information criterion (AIC; Akaike 1974), second-order AIC (AIC_C_; Sugiura 1978; Hurvich and Tsai 1989), and Bayesian information criterion (BIC Schwarz 1978).

Instead of focusing on the maximum likelihood of a model, the Bayesian approach compares the average fit of a model. This imposes a “natural” penalty for parameters, because each additional parameter introduces a dimension that must be averaged over. If that dimension introduces substantial parameter space with small likelihood, and little space that improves the likelihood, it will decrease the marginal likelihood. Thus, unlike the maximum likelihood, adding a parameter to a model can decrease the *marginal* likelihood, which ensures that more parameter-rich models are not automatically preferred.

The ratio of two marginal likelihoods gives us the factor by which the average fit of the model in the numerator is better or worse than the model in the denominator. This is called the Bayes factor (Jeffreys 1935). We can again leverage Bayes’ rule to gain more intuition for how marginal likelihoods and Bayes factors guide Bayesian model selection by writing it in terms of the posterior probability of a model, ${M_{1}}$, among ${N}$ candidate models:

(3) \begin{align*} p({M_{1}} {\,|\,} {D}) = \frac{ p({D} {\,|\,} {M_{1}}) p({M_{1}}) }{ \sum\limits_{i=1}^{{N}} p({D} {\,|\,} {M_{i}}) p({M_{i}}) }. \label{eq:bayesRuleModelProbability} \end{align*}

This shows us that the posterior probability of a model is proportional to the prior probability multiplied by the marginal likelihood of that model. Thus, the marginal likelihood is how the data update our prior beliefs about a model. As a result, it is often simply referred to as “the evidence” (MacKay 2005). If we look at the ratio of the posterior probabilities of two models,

(4) \begin{align*} \frac{ p({M_{1}} {\,|\,} {D}) }{ p({M_{2}} {\,|\,} {D}) } = \frac{ p({D} {\,|\,} {M_{1}}) }{ p({D} {\,|\,} {M_{2}}) } \times \frac{ p({M_{1}}) }{ p({M_{2}}) }, \label{eq:modelOdds} \end{align*}

we see that the Bayes factor is the factor by which the prior odds of a model is multiplied to give us the posterior odds. Thus, marginal likelihoods and their ratios give us intuitive measures of how much the data “favor” one model over another, and these measures have natural probabilistic interpretations. However, marginal likelihoods and Bayes factors do not offer a panacea for model choice. As Equation 1 shows, weighting the average likelihood by the prior causes marginal likelihoods to be inherently sensitive to the prior distributions placed on the models’ parameters. To gain more intuition about what this means and how Bayesian model choice differs from parameter estimation, let’s use a simple, albeit contrived, example of flipping a coin.

### A Coin-Flipping Example

**Figure 1.**
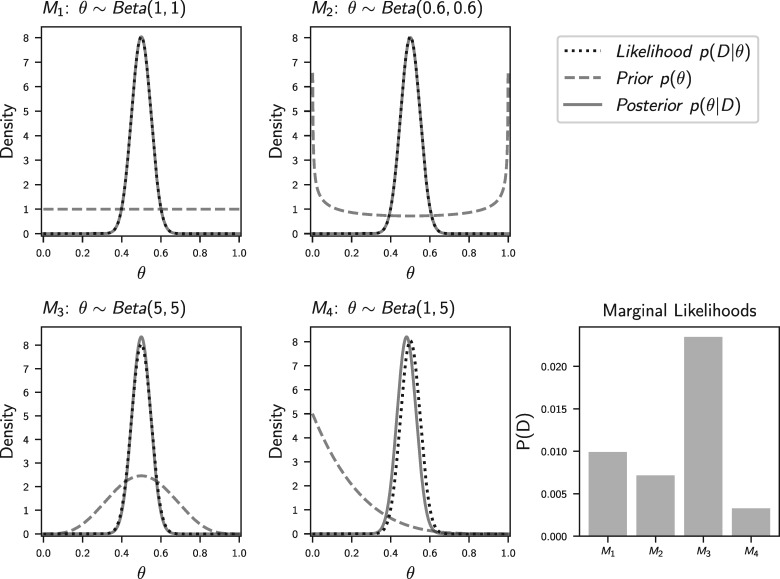
An illustration of the posterior probability densities and marginal likelihoods of the four different prior assumptions we made in our coin-flipping experiment. The data are 50 “heads” out of 100 coin flips, and the parameter, ${\theta_{}}$, is the probability of the coin landing heads side up. The binomial likelihood density function is proportional to a $\textrm{Beta}(51, 51)$ and is the same across the four different beta priors on ${\theta_{}}$ ($M_1$–$M_4$). The posterior of each model is a $\textrm{Beta}(\alpha + 50, \beta + 50)$ distribution. The marginal likelihoods ($P(D)$; the average of the likelihood density curve weighted by the prior) of the four models are compared.

Let’s assume we are interested in the probability of a coin we have not seen landing heads-side up when it is flipped (${\theta_{}}$); we refer to this as the rate of landing heads up to avoid confusion with other uses of the word probability. Our plan is to flip this coin 100 times and count the number of times it lands heads up, which we model as a random outcome from a binomial distribution. Before flipping, we decide to compare four models that vary in our prior assumptions about the probability of the coin landing heads up (Fig. 1): We assume

1. all values are equally probable (${M_{1}}$: ${\theta_{}} \sim \textrm{Beta}(1, 1)$),
2. the coin is likely weighted to land mostly “heads” or “tails” (${M_{2}}$: ${\theta_{}} \sim \textrm{Beta}(0.6, 0.6)$),
3. the coin is probably fair (${M_{3}}$: ${\theta_{}} \sim \textrm{Beta}(5.0, 5.0)$), and
4. the coin is weighted to land tails side up most of time (${M_{4}}$: ${\theta_{}} \sim \textrm{Beta}(1.0, 5.0)$).

We use beta distributions to represent our prior expectations, because the beta is a conjugate prior for the binomial likelihood function. This allows us to obtain the posterior distribution and marginal likelihood analytically.

After flipping the coin and observing that it landed heads side up 50 times, we can calculate the posterior probability distribution for the rate of landing heads up under each of our four models:

(5) \begin{align*} p({\theta_{}} {\,|\,} {D}, {M_{i}}) = \frac{ p({D} {\,|\,} {\theta_{}}, {M_{i}}) p({\theta_{}} {\,|\,} {M_{i}}) }{ p({D} {\,|\,} {M_{i}}) }. \label{eq:coinBayesRule} \end{align*}

Doing so, we see that regardless of our prior assumptions about the rate of the coin landing heads, the posterior distribution is very similar (Fig. 1). This makes sense; given we observed 50 heads out of 100 flips, values for ${\theta_{}}$ toward zero and one are extremely unlikely, and the posterior is dominated by the likelihood of values near 0.5.

Given the posterior distribution for ${\theta_{}}$ is very robust to our prior assumptions, we might assume that each of our four models explain the data similarly well. However, to compare their ability to explain the data, we need to average (integrate) the likelihood density function over all possible values of ${\theta_{}}$, weighting by the prior:

(6) \begin{align*} p({D} {\,|\,} {M_{i}}) = \int_{{\theta_{}}} p( {D} {\,|\,} {\theta_{}}, {M_{i}}) p({\theta_{}} {\,|\,} {M_{i}}) {\mathrm{d}{{\theta_{}}}}. \label{eq:coinMarginalLikelihood} \end{align*}

Looking at the plots in Figure 1, we see that the models that place a lot of prior weight on values of ${\theta_{}}$ that do not explain the data well (i.e., have small likelihood) have a much smaller marginal likelihood. Thus, even if we have very informative data that make the posterior distribution robust to prior assumptions, this example illustrates that the marginal likelihood of a model can still be very sensitive to the prior assumptions we make about the parameters.

Because of this inherent sensitivity to the priors, we have to take more care when choosing priors on the models’ parameters when our goal is to compare models versus estimating parameters. For example, in Bayesian phylogenetics, it is commonplace to use “uninformative” priors, some of which are improper (i.e., they do not integrate to one). The example above demonstrates that if we have informative data, this objective Bayesian strategy (Jeffreys 1961; Berger 2006) is defensible if our goal is to infer the posterior distribution of a model; we are hedging our bets against specifying a prior that concentrates its probability density outside of where the true value lies, and we can rely on the informative data to dominate the posterior. However, this strategy is much harder to justify if our goal is to compare marginal likelihoods among models. First of all, models with improper priors do not have a well-defined marginal likelihood and should not be used when comparing models (Baele et al. 2013b). Second, even if diffuse priors are proper, they could potentially sink the marginal likelihood of good models by placing excessive weight in biologically unrealistic regions of parameter space with low likelihood. Thus, if our goal is to leverage Bayesian model choice to learn about the processes that gave rise to our data, a different strategy is called for. One option is to take a more subjective Bayesian approach (Lad 1996; Lindley 2000; Goldstein 2006) by carefully choosing prior distributions for the models’ parameters based on existing knowledge. In the era of “big data,” one could also use a portion of their data to inform the priors, and the rest of the data for inference. Alternatively, we can use hierarchical models that allow the data to inform the priors on the parameters (e.g., Suchard et al. 2003a).

We have developed an interactive version of Figure 1 where readers can vary the parameters of the coin-flip experiment and prior assumptions to further gain intuition for marginal likelihoods (https://kerrycobb.github.io/beta-binomial-web-demo/). It’s worth noting that this pedagogical example is somewhat contrived given that the models we are comparing are simply different priors. Using the marginal likelihood to choose a prior is dubious, because the “best” prior will always be a point mass on the maximum likelihood estimate. Nonetheless, the principles of (and differences between) Bayesian parameter estimation and model choice that are illustrated by this example are directly relevant to more practical Bayesian inference settings. Now we turn to methods for approximating the marginal likelihood of phylogenetic models, where simple analytical solutions are generally not possible. Nonetheless, the same fundamental principles apply.

## Methods for Marginal Likelihood Approximation

For all but the simplest of models, the summation and integrals in Equation 1 are analytically intractable. This is particularly true for phylogenetic models, which have a complex structure containing both discrete and continuous elements. Thus, we must resort to numerical techniques to approximate the marginal likelihood.

Perhaps the simplest numerical approximation of the marginal likelihood is to draw samples of a model’s parameters from their respective prior distributions. This turns the intractable integral into a sum of the samples’ likelihoods. Because the prior weight of each sample is one in this case, the marginal likelihood can be approximated by simply calculating the average likelihood of the prior samples. Alternatively, if we have a sample of the parameters from the posterior distribution—such as one obtained from a “standard” Bayesian phylogenetic analysis via MCMC—we can again use summation to approximate the integral. In this case, the weight of each sample is the ratio of the prior density to the posterior density. As a result, the sum simplifies to the harmonic mean (HM) of the likelihoods from the posterior sample (Newton and Raftery 1994). Both of these techniques can be thought of as importance-sampling integral approximations. Whereas both provide unbiased estimates of the marginal likelihood in theory, they can suffer from very large Monte Carlo error due to the fact that the prior and posterior are often *very* divergent, with the latter usually *much* more peaked than the former due to the strong influence of the likelihood. A finite sample from the prior will often yield an underestimate of the marginal likelihood, because the region of parameter space with high likelihood is likely to be missed. In comparison, a finite sample from the posterior will almost always lead to an overestimate (Lartillot and Philippe 2006; Fan et al. 2011; Xie et al. 2011), because it will contain too few samples outside of the region of high likelihood, where the prior weight “penalizes” the average likelihood. However, Baele et al. (2016) showed that for trees with 3–6 tips and relatively simple models, the average likelihood of a very large sample from the prior (30–50 billion samples) can yield accurate estimates of the marginal likelihood.

Recent methods developed to estimate marginal likelihoods generally fall into two categories for dealing with the sharp contrast between the prior and posterior that cripples the simple approaches mentioned above. One general strategy is to turn the giant leap between the unnormalized posterior and prior into many small steps across intermediate distributions; methods that fall into this category require samples collected from the intermediate distributions. The second strategy is to turn the giant leap between the posterior and prior into a smaller leap between the posterior and a reference distribution that is as similar as possible to the posterior; many methods in this category only require samples from the posterior distribution. These approaches are not mutually exclusive (e.g., see Fan et al., 2011), but they serve as a useful way to categorize many of the methods available for approximating marginal likelihoods. In practical terms, the first strategy is computationally expensive, because samples need to be collected from each step between the posterior and prior, which is not normally part of a standard Bayesian phylogenetic analysis. The second strategy can be very inexpensive for methods that attempt to approximate the marginal likelihood using only the posterior samples collected from a typical analysis.

### Approaches that Bridge the Prior and Posterior with Small Steps

#### Path Sampling

Lartillot and Philippe (2006) introduced path sampling (PS; also called thermodynamic integration; Gelman and Meng 1998) phylogenetics to address the problem that the posterior is often dominated by the likelihood and very divergent from the prior. Rather than restrict themselves to a sample from the posterior, they collected MCMC samples from a series of distributions between the prior and posterior. Specifically, samples are taken from a series of power-posterior distributions, $ p({D} {\,|\,} {T_{}}, {\theta_{}}, {M_{}})^{\beta} p({T_{}}, {\theta_{}} {\,|\,} {M_{}})$, where the likelihood is raised to a power $\beta$. When $\beta = 1$, this is equal to the unnormalized joint posterior, which integrates to what we want to know, the marginal likelihood. When $\beta = 0$, this is equal to the joint prior distribution, which, assuming we are using proper prior probability distributions, integrates to 1. If we integrate the power posterior expectation of the derivative with respect to $\beta$ of the log power posterior over the interval (0–1) with respect to $\beta$, we get the log ratio of the normalizing constants when $\beta$ equals 1 and 0, and since we know the constant is 1 when $\beta$ is zero, we are left with the marginal likelihood. Lartillot and Philippe (2006) approximated this integral by summing over MCMC samples taken from a discrete number of $\beta$ values evenly distributed between 1 and 0.

#### Stepping-Stone Sampling

The stepping-stone (SS) method introduced by Xie et al. (2011) is similar to PS in that it also uses samples from power posteriors, but the idea is not based on approximating the integral *per se*, but by the fact that we can accurately use importance sampling to approximate the ratio of normalizing constants with respect to two pre-chosen consecutive $\beta$ values at each step between the posterior and prior. Also, Xie et al. (2011) chose the values of $\beta$ for the series of power posteriors from which to sample so that most were close to the prior (reference) distribution, rather than evenly distributed between 0 and 1. This is beneficial, because most of the change happens near the prior; the likelihood begins to dominate quickly, even at small values of $\beta$. The SS method results in more accurate estimates of the marginal likelihood with fewer steps than PS (Xie et al. 2011).

#### Generalized Stepping Stone

The most accurate estimator of marginal likelihoods available to date, the generalized stepping-stone (GSS) method, combines both strategies we are using to categorize methods by taking many small steps from a starting point (reference distribution) that is much closer to the posterior than the prior (Fan et al. 2011). Fan et al. (2011) improved upon the original SS method by using a reference distribution that, in most cases, will be much more similar to the posterior than the prior. The reference distribution has the same form as the joint prior, but each marginal prior distribution is adjusted so that its mean and variance matches the corresponding sample mean and variance of an MCMC sample from the posterior. This guarantees that the support of the reference distribution will cover the posterior.

Initially, the application of the GSS method was limited, because it required that the topology be fixed, because there was no reference distribution across topologies. However, Holder et al. (2014) introduced such a distribution on trees, allowing the GSS to approximate the fully marginalized likelihood of phylogenetic models. Baele et al. (2016) introduced additional reference distributions on trees under coalescent models. Furthermore, Wu et al. (2014) and Rannala and Yang (2017) showed that the GSS and PS methods remain statistically consistent and unbiased when the topology is allowed to vary.

Based on intuition, it may seem that GSS would fail to adequately penalize the marginal likelihood, because it would lack samples from regions of parameter space with low likelihood (i.e., it does not use samples from the prior). However, importance sampling can be used to estimate the ratio of the normalizing constant of the posterior distribution (i.e., the marginal likelihood) to the reference distribution. As long as the reference distribution is proper, such that its normalizing constant is 1.0, this ratio is equal to the marginal likelihood. As a result, any proper reference distribution that covers the same parameter space as the posterior will work. The closer the reference is to the posterior, the easier it is to estimate the ratio of their normalizing constants (and thus the marginal likelihood). In fact, at the extreme that the reference distribution matches the posterior, we can determine the marginal likelihood exactly with only a single sample, because the difference in their densities is solely due to the normalizing constant of the posterior distribution (Fan et al. 2011).

All of the methods discussed below under “Approaches that use only posterior samples” are based on this idea of estimating the unknown normalizing constant of the posterior (the marginal likelihood) by “comparing” it to a reference distribution with a known normalizing constant (or at least a known difference in normalizing constant). What is different about GSS is the use of samples from a series of power-posterior distributions in between the reference and the posterior, which make estimating the ratio of normalizing constants between each sequential pair of distributions more accurate.

The fact that PS, SS, and GSS all use samples from a series of power-posterior distributions raises some important practical questions: How many power-posterior distributions are sufficient, how should they be spaced between the reference and posterior distribution, and how many MCMC samples are needed from each? There are no simple answers to these questions, because they will vary depending on the data and model. However, one general strategy that is clearly advantageous is having most of the $\beta$ values near zero so that most of the power-posterior distributions are similar to the reference distribution (Lepage et al. 2007; Xie et al. 2011; Baele et al. 2016). Also, a practical approach to assess if the number of $\beta$ values and the number of samples from each power posterior is sufficient is to estimate the marginal likelihood multiple times (starting with different seeds for the random number generator) for each model to get a measure of variance among estimates. It is difficult to quantify how much variance is too much, but the estimates for a model should probably be within a log likelihood unit or two from each other, and the ranking among models should be consistent. It can also be useful to check how much the variance among estimates decreases after repeating the analysis with more $\beta$ values and/or more MCMC sampling from each step; a large decrease in variance suggests the sampling scheme was insufficient.

#### Sequential Monte Carlo

Another approach that uses sequential importance-sampling steps is sequential Monte Carlo (SMC), also known as particle filtering (Gordon et al. 1993; Del Moral 1996; Liu and Chen 1998). Recently, SMC algorithms have been developed for approximating the posterior distribution of phylogenetic trees (Bouchard-Côté et al. 2012; Bouchard-Côté 2014; Wang et al. 2018a). While inferring the posterior, SMC algorithms can approximate the marginal likelihood of the model “for free,” by keeping a running average of the importance-sampling weights of the trees (particles) along the way. SMC algorithms hold a lot of promise for complementing MCMC in Bayesian phylogenetics due to their sequential nature and ease with which the computations can be parallelized (Bouchard-Côté et al. 2012; Dinh et al. 2018; Fourment et al. 2018; Wang et al. 2018a). See Bouchard-Côté (2014) for an accessible treatment of SMC in phylogenetics.

Wang et al. (2018a) introduced a variant of SMC into phylogenetics that, similar to path sampling and SS, transitions from a sample from the prior distribution to the posterior across a series of distributions where the likelihood is raised to a power (annealing). This approach provides an estimator of the marginal likelihood that is unbiased from both a statistical and computational perspective. Also, their approach maintains the full state space of the model, while sampling across the power-posterior distributions, which allows them to use standard Metropolis-Hastings algorithms from the MCMC literature for the proposals used during the SMC. This should make the algorithm easier to implement in existing phylogenetic software compared with other SMC approaches that build up the state space of the model during the algorithm. Under the simulation conditions they explored, Wang et al. (2018a) showed that the annealed SMC algorithm compared favorably to MCMC and SS in terms of sampling the posterior distribution and estimating the marginal likelihood, respectively.

#### Nested Sampling

Recently, Maturana et al. (2018) introduced the numerical technique known as nested sampling (NS; Skilling 2006) to Bayesian phylogenetics. This tries to simplify the multi-dimensional integral in Equation 1 into a one-dimensional integral over the cumulative distribution function of the likelihood. The latter can be numerically approximated using basic quadrature methods, essentially summing up the area of polygons under the likelihood function. The algorithm works by starting with a random sample of parameter values from the joint prior distribution and their associated likelihood scores. Sequentially, the sample with the lowest likelihood is removed and replaced by another random sample from the prior with the constraint that its likelihood must be larger than the removed sample. The approximate marginal likelihood is a running sum of the likelihood of these removed samples with appropriate weights. Re-sampling these removed samples according to their weights yields a posterior sample at no extra computational cost. Initial assessment of NS suggest it performs similarly to GSS. As with SMC, NS seems like a promising complement to MCMC for both approximating the posterior and marginal likelihood of phylogenetic models.

### Approaches that Use Only Posterior Samples

#### Generalized Harmonic Mean

Gelfand and Dey (1994) introduced a generalized harmonic mean (GHM) estimator that uses an arbitrary normalized reference distribution, as opposed to the prior distribution used in the HM estimator, to weight the samples from the posterior. If the chosen reference distribution is more similar to the posterior than the prior (i.e., a “smaller leap” as discussed above), the GHM estimator will perform better than the HM estimator. However, for high-dimensional phylogenetic models, choosing a suitable reference distribution is very challenging, especially for tree topologies. As a result, the GHM estimator has not been used for comparing phylogenetic models. However, recent advances on defining a reference distribution on trees (Holder et al. 2014; Baele et al. 2016) makes the GHM a tenable option in phylogenetics.

As discussed above, the HM estimator is unbiased in theory, but can suffer from very large Monte Carlo error in practice. The degree to which the GHM estimator solves this problem will depend on how much more similar the chosen reference distribution is to the posterior compared with the prior. Knowing whether it is similar enough in practice will be difficult without comparing the estimates to other unbiased methods with much smaller Monte Carlo error (e.g., GSS, PS, or SMC).

#### Inflated-Density Ratio

The inflated-density ratio (IDR) estimator solves the problem of choosing a reference distribution by using a perturbation of the posterior density; essentially the posterior is “inflated” from the center by a known radius (Petris and Tardella 2007; Arima and Tardella 2012; Arima and Tardella 2014). As one might expect, the radius must be chosen carefully. The application of this method to phylogenetics has been limited by the fact that all parameters must be unbounded; any parameters that are bounded (e.g., must be positive) must be re-parameterized to span the real number line, perhaps using log transformation. As a result, this method cannot be applied directly to MCMC samples collected by popular Bayesian phylogenetic software packages. Nonetheless, the IDR estimator has recently been applied to phylogenetic models (Arima and Tardella 2014), including in settings where the topology is allowed to vary (Wu et al. 2014). Initial applications of the IDR are very promising, demonstrating comparable accuracy to methods that sample from power-posterior distributions, while avoiding such computation (Arima and Tardella 2014; Wu et al. 2014). Currently, however, the IDR has only been used on relatively small data sets and simple models of character evolution. More work is necessary to determine whether the promising combination of accuracy and computational efficiency holds for large data sets and rich models.

#### Partition-Weighted Kernel

Recently, Wang et al. (2018b) introduced the partition weighted kernel (PWK) method of approximating marginal likelihoods. This approach entails partitioning parameter space into regions within which the posterior density is relatively homogeneous. Given the complex structure of phylogenetic models, it is not obvious how this would be done. As of yet, this method has not been used for phylogenetic models. However, for simulations of mixtures of bivariate normal distributions, the PWK outperforms the IDR estimator (Wang et al. 2018b). Thus, the method holds promise if it can be adapted to phylogenetic models.

## Uses of Marginal Likelihoods

The application of marginal likelihoods to compare phylogenetic models is rapidly gaining popularity. Rather than attempt to be comprehensive, below we highlight examples that represent some of the diversity of questions being asked and the insights that marginal likelihoods can provide about our data and the evolutionary processes giving rise to them.

### Comparing Partitioning Schemes

One of the earliest applications of marginal likelihoods in phylogenetics was to choose among ways of assigning models of substitution to different subsets of aligned sites. This became important when phylogenetics moved beyond singe-locus trees to concatenated alignments of several loci. Mueller et al. (2004), Nylander et al. (2004), and Brandley et al. (2005) used Bayes factors calculated from HM estimates of marginal likelihoods to choose among different strategies for partitioning aligned characters to substitution models. All three studies found that the model with the most subsets was strongly preferred. Nylander et al. (2004) also showed that removing parameters for which the data seemed to have little influence decreased the HM estimates of the marginal likelihood, suggesting that the HM estimates might favor over-parameterized models. These findings could be an artifact of the tendency of the HM estimator to overestimate marginal likelihoods and thus underestimate the “penalty” associated with the prior weight of additional parameters. However, Brown and Lemmon (2007) showed that for simulated data, HM estimates of Bayes factors can have a low error rate of over-partitioning an alignment.

Fan et al. (2011) showed that, again, the HM estimator strongly favors the most partitioned model for a four-gene alignment from cicadas (12 subsets partitioned by gene and codon position). However, the marginal likelihoods estimated via the GSS stone method favor a much simpler model (three subsets partitioned by codon position). This demonstrates how the HM method fails to penalize the marginal likelihood for the weight of the prior when applied to finite samples from the posterior. It also suggests that relatively few, well-assigned subsets can go a long way to explain the variation in substitution rates among sites.

Baele and Lemey (2013) compared the marginal likelihoods of alternative partitioning strategies (in combination with either strict or relaxed-clock models) for an alignment of whole mitochondrial genomes of carnivores. They used the HM, stabilized HM (Newton and Raftery 1994), PS, and SS estimators. For all 41 models they evaluated, both HM estimators returned much larger marginal likelihoods than PS and SS, again suggesting these estimators based solely on the posterior sample are unable to adequately penalize the models. They also found that by allowing the sharing of information among partitions via hierarchical modeling (Suchard et al. 2003a), the model with the largest PS and SS-estimated marginal likelihood switched from a codon model to a nucleotide model partitioned by codon position. This demonstrates the sensitivity of marginal likelihoods to prior assumptions.

### Comparing Models of Character Substitution

Lartillot and Philippe (2006) used PS to compare models of amino-acid substitution. They found that the HM estimator favored the most parameter rich model for all five data sets they explored, whereas the PS estimates favored simpler models for three of the data sets. This again demonstrates that accurately estimated marginal likelihoods can indeed “penalize” for over-parameterization of phylogenetic models. More importantly, this work also revealed that modeling heterogeneity in amino acid composition across sites of an alignment better explains the variation in biological data.

### Comparing “Relaxed Clock” Models

Lepage et al. (2007) used PS to approximate Bayes factors comparing various “relaxed-clock” phylogenetic models for three empirical data sets. They found that models in which the rate of substitution evolves across the tree (autocorrelated rate models) better explain the empirical sequence alignments they investigated than models that assume the rate of substitution on each branch is independent (uncorrelated rate models). This provides insight into how the rate of evolution evolves through time.

Baele et al. (2013b) demonstrated that modeling among-branch rate variation with a lognormal distribution tends to explain mammalian sequence alignments better than using an exponential distribution. They used marginal likelihoods (PS and SS estimates) and Bayesian model averaging to compare the fit of lognormally and exponentially distributed priors on branch-specific rates of nucleotide substitution (i.e., relaxed clocks) for almost 1000 loci from 12 mammalian species. They found that the lognormal relaxed-clock was a better fit for almost 88% of the loci. Baele et al. (2012) also used marginal likelihoods to demonstrate the importance of using sampling dates when estimating time-calibrated phylogenetic trees. They used PS and SS methods to estimate the marginal likelihoods of strict and relaxed-clock models for sequence data of herpes viruses. They found that when the dates the viruses were sampled were provided, a strict molecular clock was the best fit model, but when the dates were excluded, relaxed-clock models were strongly favored. Their findings show that using information about the ages of the tips can be critical for accurately modeling processes of evolution and inferring evolutionary history.

### Comparing Demographic Models

Baele et al. (2012) used the PS and SS estimators for marginal likelihoods to compare the fit of various demographic models to the HIV-1 group M data of Worobey et al. (2008), and Methicillin-resistant *Staphylococcus aureus* (MRSA) data of Gray et al. (2011). They found that a flexible, non-parametric model that enforces no particular demographic history is a better explanation of the HIV and MRSA sequence data than exponential and logistic population growth models. This suggests that traditional parametric growth models are not the best predictors of viral and bacterial epidemics.

### Measuring Phylogenetic Information Content Across Genomic Data Sets

Not only can we use marginal likelihoods to learn about evolutionary models, but we can also use them to learn important lessons about our data. Brown and Thomson (2017) explored six different genomic data sets that were collected to infer phylogenetic relationships within Amniota. For each locus across all six data sets, they used the SS method (Xie et al. 2011) to approximate the marginal likelihood of models that included or excluded a particular branch (bipartition) in the amniote tree. This allowed Brown and Thomson (2017) to calculate, for each gene, Bayes factors as measures of support for or against particular relationships, some of which are uncontroversial (e.g., the monophyly of birds) and others contentious (e.g., the placement of turtles).

Such use of marginal likelihoods to compare topologies, or constraints on topologies, raises some interesting questions. Bergsten et al. (2013) showed that using Bayes factors for topological tests can result in strong support for a constrained topology over an unconstrained model for reasons other than the data supporting the branch (bipartition) being constrained. This occurs when the data support other branches in the tree that make the constrained branch more likely to be present just by chance, compared with a diffuse prior on topologies. This is not a problem with the marginal likelihoods (or their estimates), but rather how we interpret the results of the Bayes factors; if we want to interpret it as support for a particular relationship, we have to be cognizant of the topology space we are summing over under both models. Brown and Thomson (2017) tried to limit the effect of this issue by constraining all “uncontroversial” bipartitions when they calculate the marginal likelihoods of models with and without a particular branch, essentially enforcing an informative prior across topologies under both models.

Brown and Thomson’s (2017) use of marginal likelihoods allowed them to reveal a large degree of variation among loci in support for and against relationships that was masked by the corresponding posterior probabilities estimated by MCMC. Furthermore, they found that a small number of loci can have a large effect on the tree and associated posterior probabilities of branches inferred from the combined data. For example, they showed that including or excluding just two loci out of the 248 locus data set of (Chiari et al. 2012) resulted in a posterior probability of 1.0 in support of turtles either being sister to crocodylians or archosaurs (crocodylians and birds), respectively. By using marginal likelihoods of different topologies, Brown and Thomson (2017) were able to identify these two loci as putative paralogs due to their strikingly strong support for turtles being sister to crocodylians. This work demonstrates how marginal likelihoods can simultaneously be used as a powerful means of controlling the quality of data in “phylogenomics”, while informing us about the evolutionary processes that gave rise to our data.

Furthermore, Brown and Thomson (2017) found that the properties of loci commonly used as proxies for the reliability of phylogenetic signal (rate of substitution, how “clock-like” the rate is, base composition heterogeneity, amount of missing data, and alignment uncertainty) were poor predictors of Bayes factor support for well-established amniote relationships. This suggests these popular rules of thumb are not useful for identifying “good” loci for phylogenetic inference.

### Phylogenetic Factor Analysis

The goal of comparative biology is to understand the relationships among a potentially large number of phenotypic traits across organisms. To do so correctly, we need to account for the inherent shared ancestry underlying all life (Felsenstein 1985). A lot of progress has been made for inferring the relationship between pairs of phenotypic traits as they evolve across a phylogeny, but a general and efficient solution for large numbers of continuous and discrete traits has remained elusive. Tolkoff et al. (2018) introduced Bayesian factor analysis to a phylogenetic framework as a potential solution. Phylogenetic factor analysis works by modeling a small number of unobserved (latent) factors that evolve independently across the tree, which give rise to the large number of observed continuous and discrete phenotypic traits. This allows correlations among traits to be estimated, without having to model every trait as a conditionally independent process.

The question that immediately arises is, what number of factors best explains the evolution of the observed traits? To address this, Tolkoff et al. (2018) use PS to approximate the marginal likelihood of models with different numbers of traits. To do so, they extend the PS method to handle the latent variables underlying the discrete traits by softening the thresholds that delimit the discrete character states across the series of power posteriors. This new approach leverages Bayesian model comparison via marginal likelihoods to learn about the processes governing the evolution of multidimensional phenotypes.

### Comparing Phylogeographic Models

Phylogeographers are interested in explaining the genetic variation within and among species across a landscape. As a result, we are often interested in comparing models that include various combinations of micro- and macroevolutionary processes and geographic and ecological parameters. Deriving the likelihood function for such models is often difficult and, as a result, model choice approaches that use approximate-likelihood Bayesian computation (ABC) are often used.

At the forefront of generalizing phylogeographic models is an approach that is referred to as iDDC, which stands for integrating distributional, demographic, and coalescent models (Papadopoulou and Knowles 2016). This approach simulates data under various phylogeographical models upon proxies for habitat suitability derived from species distribution models. To choose the model that best explains the empirical data, this approach compares the marginal densities of the models approximated with general linear models (ABC-GLM; Leuenberger and Wegmann 2010), and $P$ values derived from these (He et al. 2013; Massatti and Knowles 2016; Bemmels et al. 2016; Papadopoulou and Knowles 2016; Knowles and Massatti 2017). This approach is an important step forward for bringing more biological realism into phylogeographical models. However, our findings below (see section on “approximate-likelihood approaches” below) show that the marginal GLM density fitted to a truncated region of parameter space should not be interpreted as a marginal likelihood of the full model. Thus, these methods should be seen as a useful exploration of data, rather than rigorous hypothesis tests. Because ABC-GLM marginal densities fail to penalize parameters for their prior weight in regions of low likelihood, these approaches will likely be biased toward over-parameterized phylogeographical models. Nonetheless, knowledge of this bias can help guide interpretations of results.

### Species Delimitation

Calculating the marginal probability of sequence alignments (Grummer et al. 2013) and single-nucleotide polymorphisms (Leaché et al. 2014) under various multi-species coalescent models has been used to estimate species boundaries. By comparing the marginal likelihoods of models that differ in how they assign individual organisms to species, systematists can calculate Bayes factors to determine how much the genetic data support different delimitations. Using simulated data, Grummer et al. (2013) found that marginal likelihoods calculated using PS and SS methods outperformed HM estimators at identifying the true species delimitation model. Marginal likelihoods seem better able to distinguish some species delimitation models than others. For example, models that lump species together or reassign samples into different species produce larger marginal likelihood differences versus models that split populations apart (Grummer et al. 2013; Leaché et al. 2014). Current implementations of the multi-species coalescent assume strict models of genetic isolation, and oversplitting populations that exchange genes creates a difficult Bayesian model comparison problem that does not include the correct model (Leaché et al. 2018a,b).

Species delimitation using marginal likelihoods in conjunction with Bayes factors has some advantages over alternative approaches. The flexibility of being able to compare non-nested models that contain different numbers of species, or different species assignments, is one key advantage. The methods also integrate over gene trees, species trees, and other model parameters, allowing the marginal likelihoods of delimitations to be compared without conditioning on any parameters being known. Marginal likelihoods also provide a natural way to rank competing models while automatically accounting for model complexity (Baele et al. 2012). Finally, it is unnecessary to assign prior probabilities to the alternative species delimitation models being compared. The marginal likelihood of a delimitation provides the factor by which the data update our prior expectations, regardless of what that expectation is (Equation 3). As multi-species coalescent models continue to advance, using the marginal likelihoods of delimitations will continue to be a powerful approach to learning about biodiversity.

## Alternatives to Marginal Likelihoods for Bayesian Model Choice

### Bayesian Model Averaging

Bayesian model averaging provides a way to avoid model choice altogether. Rather than infer the parameter of interest (e.g., the topology) under a single “best” model, we can incorporate uncertainty by averaging the posterior over alternative models. In situations where model choice is not the primary goal, and the parameter of interest is sensitive to which model is used, model averaging is arguably the best solution from a Bayesian standpoint. Nonetheless, when we jointly sample the posterior across competing models, we can use the posterior sample for the purposes of model choice. The frequency of samples from each model approximates its posterior probability, which can be used to approximate Bayes factors among models. Note, this approach is still based on marginal likelihoods—the marginal likelihood is how the data inform the model posterior probabilities, and the Bayes factor is simply a ratio of marginal likelihoods (Equations 3 and 4). However, by sampling across models, we can avoid calculating the marginal likelihoods directly.

Algorithms for sampling across models include reversible-jump MCMC (Green 1995), Gibbs sampling (Neal 2000), Bayesian stochastic search variable selection (George and McCulloch 1993; Kuo and Mallick 1998), and approximations of reversible-jump (Jones et al. 2015). In fact, the first application of Bayes factors for phylogenetic model comparison was performed by Suchard et al. (2001) via reversible-jump MCMC. This technique was also used in Bayesian tests of phylogenetic incongruence/recombination (Suchard et al. 2003b; Minin et al. 2005). In terms of selecting the correct “relaxed-clock” model from simulated data, Baele et al. (2013b) and Baele and Lemey (2014) showed that model-averaging performed similarly to the PS and SS marginal likelihood estimators.

There are a couple of limitations for these approaches. First, a Bayes factor that includes a model with small posterior probability will suffer from Monte Carlo error. For example, unless a very large sample from the posterior is collected, some models might not be sampled at all. A potential solution to this problem is adjusting the prior probabilities of the models such that none of their posterior probabilities are very small (Carlin and Chib 1995; Suchard et al. 2005). Second, and perhaps more importantly, for these numerical algorithms to be able to “jump” among models, the models being sampled need to be similar. Whereas the first limitation is specific to using model averaging to estimate Bayes factors, the second problem is more general.

In comparison, with estimates of marginal likelihooods in hand, we can compare any models, regardless of how different they are in terms of parameterization or relative probability. Alternatively, Lartillot and Philippe (2006) introduced a method of using path sampling to directly approximate the Bayes factor between two models that can be highly dissimilar. Similarly, Baele et al. (2013a) extended the SS approach of Xie et al. (2011) to do the same.

### Measures of Predictive Performance

Another, albeit not an unrelated, way to compare models is based on their predictive power, with the idea that we should prefer the model that best predicts future data. There are many approaches to do this, but they are all centered around measuring the predictive power of a model using the marginal probability of new data (${D}'$) given our original data (${D}$),

(7) \begin{align*} p({D}' {\,|\,} {M_{}}, {D}) = \sum\limits_{{T_{}}} \int_{{\theta_{}}} p({D}' {\,|\,} {T_{}}, {\theta_{}}, {M_{}}) p({T_{}}, {\theta_{}} {\,|\,} {M_{}}, {D}) {\mathrm{d}{{\theta_{}}}}, \label{eq:marginalPredictiveLikelihood} \end{align*}

which we will call the marginal posterior predictive likelihood. This has clear parallels to the marginal likelihood (see Equation 1), with one key difference: We condition on our knowledge of the original data, so that the average of the likelihood of the new data is now weighted by the *posterior* distribution rather than the prior. Thus, in situations where our data are informative and dominate the posterior distribution under each model, the marginal posterior predictive likelihood should be much less sensitive than the marginal likelihood to the prior distributions used for the models’ parameters.

Whether one should favor a posterior-predictive perspective or marginal likelihoods will depend on the goals of a particular model-choice exercise and whether the prior is the appropriate penalty for adding parameters to a model. Regardless, posterior predictive measures of model fit are a valuable complement to marginal likelihoods. Methods based on the marginal posterior predictive likelihood tend to be labeled with one of two names depending on the surrogate they use for the “new” data (${D}'$): (1) *cross-validation methods* partition the data under study into a training (${D}$) and testing (${D}'$) data set, whereas (2) *posterior-predictive methods* generate ${D}'$ via simulation.

#### Cross-Validation Methods

With joint samples of parameter values from the posterior (conditional on the training data ${D}$), we can easily get a Monte Carlo approximation of the marginal posterior predictive likelihood (Equation 7) by simply taking the average probability of the testing data across the posterior samples of parameter values:

(8) \begin{align*} p({D}' {\,|\,} {M_{}}, {D}) \simeq \frac{1}{{n}} \sum\limits_{i-1}^{{n}} p({D}' {\,|\,} {T_{i}}, {\theta_{i}}, {M_{}}), \label{eq:approxMarginalPredictiveLikelihood} \end{align*}

where ${n}$ is the number of samples from the posterior under model ${M_{}}$. Lartillot et al. (2007) used this approach to show that a mixture model that accommodates among-site heterogeneity in amino acid frequencies is a better predictor of animal sequence alignments than standard amino-acid models. This corroborated the findings of Lartillot and Philippe (2006) based on PS estimates of marginal likelihoods. Lewis et al. (2014) introduced a leave-one-out cross-validation approach to phylogenetics called the conditional predictive ordinates method (Geisser 1980; Gelfand et al. 1992; Chen et al. 2000). This method leaves one site out of the alignment to serve as the testing data to estimate $p({D}' {\,|\,} {M_{}}, {D})$, which is equal to the posterior harmonic mean of the site likelihood (Chen et al. 2000). Summing the log of this value across all sites yields what is called the log pseudomarginal likelihood (LPML). Lewis et al. (2014) compared the estimated LPML to SS estimates of the marginal likelihood for selecting among models that differed in how they partitioned sites across a concatenated alignment of four genes from algae. The LPML favored a 12-subset model (partitioned by gene and codon position) as opposed to the three-subset model (partitioned by codon) preferred by marginal likelihoods. This difference could reflect the lesser penalty against additional parameters imposed by the weight of the posterior (Equation 7) versus the prior (Equation 1).

#### Posterior-Predictive Methods

Alternatively, we can take a different Monte Carlo approach to Equation 7 and sample from $p({D}' {\,|\,} {M_{}}, {D})$ by simulating data sets. For each posterior sample of the parameter values (conditional on all the data under study) we can simply simulate a new data set based on those parameter values. We can then compare the observed data (${D}$) to the sample of simulated data sets (${D}'$) from the posterior predictive distribution. In all but the most trivial phylogenetic data sets, it is not practical to compare the counts of site patterns directly, because there are too many possible patterns (e.g., four raised to the power of the number of tips for DNA data). Thus, we have to tolerate some loss of information by summarizing the data in some way to reduce the dimensionality. Once a summary statistic is chosen, perhaps the simplest way to evaluate the fit of the model is to approximate the posterior predictive *P* value by finding the percentile of the statistic from the observed data out of the values of the statistic calculated from the simulated data sets (Rubin 1984; Gelfand et al. 1992). Bollback (2002) explored this approach for phylogenetic models using simulated data, and found that a simple JC69 model (Jukes and Cantor 1969) was often rejected for data simulated under more complex K2P (Kimura 1980) and GTR (Tavaré et al. 1997) models. Lartillot et al. (2007) also used this approach to corroborate their findings based on marginal likelihoods (Lartillot and Philippe 2006) and cross validation that allowing among-site variation in amino acid composition (i.e., the CAT model) leads to a better fit.

One drawback of the posterior predictive *P* value is that it rewards models with large posterior predictive variance (Lewis et al. 2014). In other words, a model that produces a broad enough distribution of data sets can avoid the observed data falling into one of the tails. The method of Gelfand and Ghosh (1998) (GG) attempts to solve this problem by balancing the tradeoff between posterior predictive variance and goodness-of-fit. Lewis et al. (2014) introduced the GG method into phylogenetics and compared it to cross-validation (LPML) and SS estimates of marginal likelihoods for selecting among models that differed in how they partitioned the sites of a four-gene alignment of algae. Similar to LPML, the GG method preferred the model with most subsets (12; partitioned by gene and codon position), in contrast to the marginal likelihood estimates, which favored the model partitioned by codon position (three subsets). Again, this difference could be due to the lesser penalty against parameters imposed by the weight of the posterior (Equation 7) versus the prior (Equation 1).

### Approximate-Likelihood Approaches

Approximate-likelihood Bayesian computation approaches Tavaré et al. 1997; Beaumont et al. 2002) have become popular in situations where it is not possible (or undesirable) to derive and compute the likelihood function of a model. The basic idea is simple: by generating simulations under the model, the fraction of times that we generate a simulated data set that matches the observed data is a Monte Carlo approximation of the likelihood. Because simulating the observed data exactly is often not possible (or extremely unlikely), simulations “close enough” to the observed data are counted, and usually a set of insufficient summary statistics are used in place of the data. Whether a simulated data set is “close enough” to count is formalized as whether or not it falls within a zone of tolerance around the empirical data.

This simple approach assumes the likelihood within the zone of tolerance is constant. However, this zone usually needs to be quite large for computational tractability, so this assumption does not hold. Leuenberger and Wegmann (Leuenberger and Wegmann 2010) proposed fitting a general linear model (GLM) to approximate the likelihood within the zone of tolerance. With the GLM in hand, the marginal likelihood of the model can simply be approximated by the marginal density of the GLM.

The accuracy of this estimator has not been assessed. However, there are good theoretical reasons to be skeptical of its accuracy. Because the GLM is only fit within the zone of tolerance (also called the “truncated prior”), it cannot account for the weight of the prior on the marginal likelihood outside of this region. Whereas the posterior distribution usually is not strongly influenced by regions of parameter space with low likelihood, the marginal likelihood very much is. By not accounting for prior weight in regions of parameter space outside the zone of tolerance, where the likelihood is low, we predict this method will not properly penalize models and tend to favor models with more parameters.

To test this prediction, we assessed the behavior of the ABC-GLM method on 100 data sets simulated under the simplest possible phylogenetic model: two DNA sequences separated by a single branch along which the sequence evolved under a Jukes-Cantor model of nucleotide substitution (Jukes and Cantor 1969). The simulated sequences were 10,000 nucleotides long, and the prior on the only parameter in the model, the length of the branch, was a uniform distribution from 0.0001 to 0.1 substitutions per site. For such a simple model, we used quadrature integration to calculate the marginal likelihood for each simulated alignment of two sequences. Integration using 1000 and 10,000 steps and rectangular and trapezoidal quadrature rules all yielded identical values for the log marginal likelihood to at least five decimal places for all 100 simulated data sets, providing a very precise proxy for the true values. We used a sufficient summary statistic, the proportion of variable sites, for ABC analyses. However, the ABC-GLM and quadrature marginal likelihoods are not directly comparable, because the marginal probability of the proportion of variable sites versus the site pattern counts will be on different scales that are data set dependent. So, we compare the ratio of marginal likelihoods (i.e., Bayes factors) comparing the correct branch-length model [branch length $\sim$ uniform(0.0001, 0.1)] to a model with a prior approximately twice as broad [branch length $\sim$ uniform(0.0001, 0.2)]. As we noted in our coin-flipping example, using marginal likelihoods to compare priors is dubious, and we do not advocate selecting priors in this way. However, in this case, comparing the marginal likelihood under these two priors is useful, because it allows us to directly test our prediction that the ABC-GLM method will not be able to correctly penalize the marginal likelihood for the additional parameter space under the broader prior.

**Figure 2.**
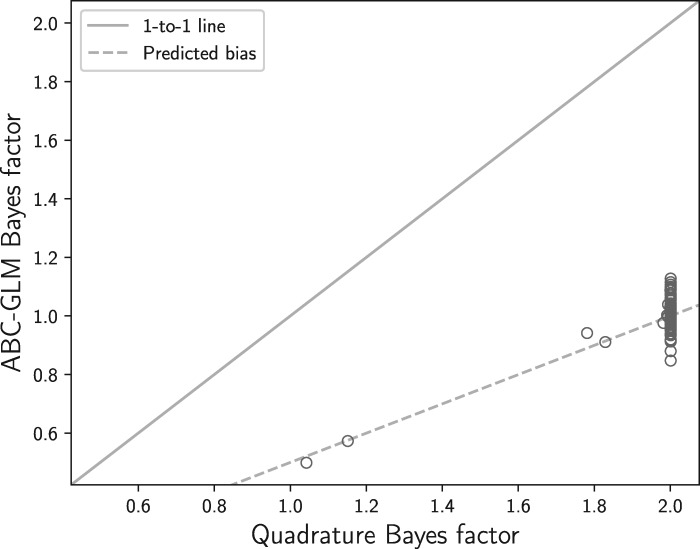
A comparison of the approximate-likelihood Bayesian computation general linear model (ABC-GLM) estimator of the marginal likelihood (Leuenberger and Wegmann 2010) to quadrature integration approximations (Xie et al. 2011) for 100 simulated data sets. We compared the ratio of the marginal likelihood (Bayes factor) comparing the correct branch-length model [branch length $\sim$ uniform(0.0001, 0.1)] to a model with a broader prior on the branch length [branch length $\sim$ uniform(0.0001, 0.2)]. The solid line represents perfect performance of the ABC-GLM estimator (i.e., matching the “true” value of the Bayes factor). The dashed line represents the expected Bayes factor when failing to penalize for the extra parameter space (branch length 0.1 to 0.2) with essentially zero likelihood. Quadrature integration with 1000 and 10,000 steps using the rectangular and trapezoidal rule produced identical values of log marginal likelihoods to at least five decimal places for all 100 simulated data sets.

**Figure A.1.**
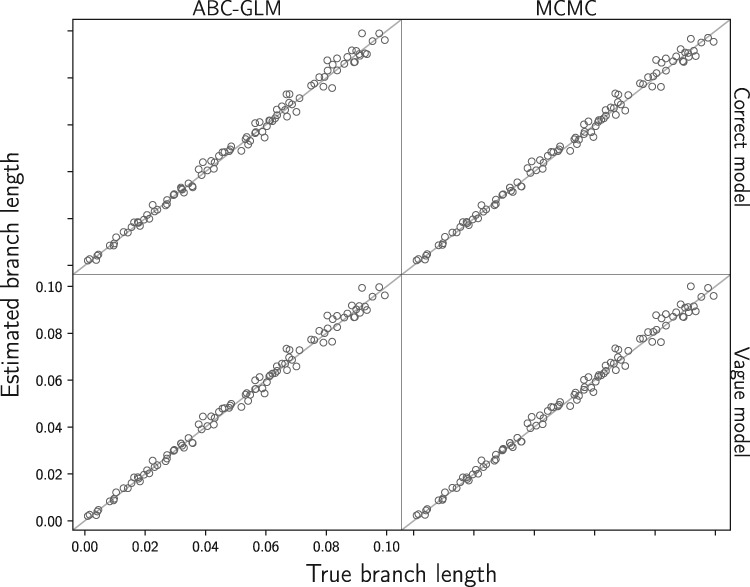
A comparison of the true branch length separating each pair of simulated sequences to the branch length estimated by ABC-GLM and full-likelihood MCMC under the correct branch-length model (branch length $\sim$ uniform(0.0001, 0.1)) and the vague model (branch length $\sim$ uniform(0.0001, 0.1)).

This very simple model is a good test of the ABC-GLM marginal likelihood estimator for several reasons. The use of a sufficient statistic for a finite, one-dimensional model makes ABC nearly equivalent to a full-likelihood Bayesian method (Fig. A.1). Thus, this is a “best-case scenario” for the ABC-GLM approach. Also, we can use quadrature integration for very good proxies for the true Bayes factors. Lastly, the simple scenario gives us some analytical expectations for the behavior of ABC-GLM. If it cannot penalize the marginal likelihood for the additional branch length space in the model with the broader prior, the Bayes factor should be off by a factor of approximately 2, or more precisely $(0.2-0.0001) / (0.1-0.0001)$. As shown in Figure 2, this is exactly what we find. This confirms our prediction that the ABC-GLM approach cannot average over regions of parameter space with low likelihood and thus will be biased toward favoring models with more parameters. Given that the GLM approximation of the likelihood is only fit within a subset of parameter space with high likelihood, which is usually a *very* small region of a model, the marginal of the GLM should not be considered a marginal likelihood of the model. We want to emphasize that our findings in no way detract from the usefulness of ABC-GLM for parameter estimation.

Full details of these analyses, which were all designed atop the DendroPy phylogenetic API (version 4.3.0 commit 72ce015) (Sukumaran and Holder 2010), can be found in Appendix A, and all of the code to replicate our results is freely available at https://github.com/phyletica/abc-glm-marginal-test.

## Discussion

### Promising Future Directions

As Bayesian phylogenetics continues to explore more complex models of evolution, and data sets continue to get larger, accurate and efficient methods of estimating marginal likelihoods will become increasingly important. Thanks to substantial work in recent years, robust methods have been developed, such as the GSS approach (Fan et al. 2011). However, these methods are computationally demanding as they have to sample likelihoods across a series of power-posterior distributions that are not useful for parameter estimation. Recent work has introduced promising methods to estimate marginal likelihoods solely from samples from the posterior distribution. However, these methods remain difficult to apply to phylogenetic models, and their performance on rich models and large data sets remains to be explored.

Promising avenues for future research on methods for estimating marginal likelihoods of phylogenetic models include continued work on reference distributions that are as similar to the posterior as possible, but easy to formulate and use. This would improve the performance and applicability of the GSS and derivations of the GHM approach. Currently, the most promising method that works solely from a posterior sample is IDR. Making this method easier to apply to phylogenetic models and implementing it in popular Bayesian phylogenetic software packages, such as RevBayes (Höhna et al. 2016) and BEAST (Suchard et al. 2018; Bouckaert et al. 2014) would be very useful, though nontrivial.

Furthermore, nested sampling and sequential Monte Carlo (SMC) are exciting numerical approaches to Bayesian phylogenetics. These methods essentially use the same amount of computation to both sample from the posterior distribution of phylogenetic models and provide an approximation of the marginal likelihood. Both approaches are relatively new to phylogenetics, but hold a lot of promise for Bayesian phylogenetics generally and model comparison via marginal likelihoods specifically.

### A Fundamental Challenge of Bayesian Model Choice

While the computational challenges to approximating marginal likelihoods are very real and will provide fertile ground for future research, it is often easy to forget about a fundamental challenge of Bayesian model choice. This challenge becomes apparent when we reflect on the differences between Bayesian model choice and parameter estimation. The posterior distribution of a model, and associated parameter estimates, are informed by the likelihood function (Equation 2), whereas the posterior probability of that model is informed by the *marginal* likelihood (Equation 3). When we have informative data, the posterior distribution is dominated by the likelihood, and as a result our parameter estimates are often robust to prior assumptions we make about the parameters. However, when comparing models, we need to assess their overall ability to predict the data, which entails averaging over the entire parameter space of the model, not just the regions of high likelihood. As a result, marginal likelihoods and associated model choices can be very sensitive to priors on the *parameters* of each model, even when the data are very informative (Fig. 1). This sensitivity to prior assumptions about parameters is inherent to Bayesian model choice based on marginal likelihoods (i.e., Bayes factors and Bayesian model averaging). However, other Bayesian model selection approaches, such as cross-validation and posterior-predictive methods, will be less sensitive to prior assumptions. Regardless, the results of any application of Bayesian model selection should be accompanied by an assessment of the sensitivity of those results to the priors placed on the models’ parameters.

## Conclusions

Marginal likelihoods are intuitive measures of model fit that are grounded in probability theory. As a result, they provide us with a coherent way of gaining a better understanding about how evolution proceeds as we accrue biological data. We highlighted how marginal likelihoods of phylogenetic models can be used to learn about evolutionary processes and how our data inform our models. Because shared ancestry is a fundamental property of life, the use of marginal likelihoods of phylogenetic models promises to continue to advance biology.

## Appendix

### Methods for Assessing Performance of ABC-GLM Estimator

We set up a simple scenario for assessing the performance of the method for estimating marginal likelihoods based on approximating the likelihood function with a general linear model (GLM) fitted to posterior samples collected via ABC (Leuenberger and Wegmann 2010); hereforth referred to as ABC-GLM. The scenario is a DNA sequence, 10,000-nucleotides in length, that evolves along a branch according to a Jukes–Cantor continuous-time Markov chain (CTMC) model of nucleotide substitution (Jukes and Cantor 1969). Because the Jukes–Cantor model forces the relative rates of change among the four nucleotides and the equilibrium nucleotide frequencies to be equal, there is only a single parameter in the model, the length of the branch, and the direction of evolution along the branch does not matter.

#### Simulating Data Sets

We simulated 100 data sets under this model by

1. drawing 10,000 nucleotides of the “ancestral” sequence from their equilibrium frequencies ($\frac{1}{4}$),
2. drawing a branch length $\sim$ uniform(0.0001, 0.1), and
3. evolving the sequence along the branch according to the Jukes–Cantor CTMC model to get the “descendant” sequence.

This was done using the DendroPy phylogenetic API (version 4.3.0 commit 72ce015) (Sukumaran and Holder 2010).

#### Calculating “True” Bayes Factors

For each data set, we used quadrature approaches to approximate the marginal likelihood by integrating the posterior density over the branch length prior. We did this for two models:

1. the correct model [branch length $\sim$ uniform(0.0001, 0.1)], and
2. a model with a branch length prior slightly more than twice as broad [branch length $\sim$ uniform(0.0001, 0.2)], which we refer to as the “vague model”.

For both models and for each data set we used the rectangular and trapezoidal quadrature rules with 1000 and 10,000 steps (i.e., four approximations of the marginal likelihood for each data set under each model). Across all 100 data sets and both models, all four approximations were identical to at least five decimal places. For each data set, we calculated the log Bayes factor comparing the correct model to the vague model.

#### Approximate-Likelihood Bayesian Computation

To collect an approximate posterior sample from the correct model for a data set, we first calculated the proportion of variable sites (${Pvar_{}}$) between the two sequences. Next, we simulated 50,000 data sets under the correct model, calculated ${Pvar_{}}$ for each of them, and retained the 1000 samples with the values of ${Pvar_{}}$ closest to that calculated from the data. Lastly, we used ABCtoolbox version 1.1 Wegmann et al. (2010) to fit a GLM to the retained samples and calculate the marginal density of the GLM, using a bandwidth of 0.002. We did the same to obtain an ABC-GLM estimate of the marginal density for the vague model with two differences: (1) we drew the branch length for each prior sample from the vague prior [branch length $\sim$ uniform(0.0001, 0.2)], and (2) to maintain the same expected tolerance under both models, we simulated 100,000 data sets under the vague model (retaining the 1000 samples closest to the ${Pvar_{}}$ of the data).

For each data set, we calculated the log Bayes factor from the GLM marginal densities of the correct and vague model, and compared the ABC-GLM-estimated Bayes factor to the “true” Bayes factor calculated via quadrature integration (Fig. 2).

#### Full-Likelihood Markov Chain Monte Carlo Analyses

One goal of the simplicity of the above model is that the additional approximation of the ABC approach would be limited. All numerical Bayesian analyses, based on full or approximate likelihoods, suffer from Monte Carlo error associated with approximating the posterior with a finite number of samples. Approximate-likelihood methods usually suffer from two additional sources of approximation: (1) the full data are replaced with insufficient summary statistics, and (2) samples are retained that do not exactly match the data or summary statistics (i.e., the “tolerance” of ABC). In our analyses described above, we avoided the former source of error by using a sufficient statistic. We hoped to minimize the latter source of error by evaluating many samples from a one-dimensional model with finite bounds; we also kept this source of error approximately equal for both models by sampling in proportion to the width of the model.

To verify that the error introduced by the tolerance of the ABC analyses was minimal, we compared the branch length estimates to those estimated by full-likelihood Markov chain Monte Carlo (MCMC). For each data set, under both models, we ran a chain for 10,000 generations, sampling every 10 generations. All chains appeared to reach stationarity by the first sample (10th generation). We plotted the branch length estimated via ABC-GLM and MCMC under both the true and vague models against the true branch lengths. The results of all four analyses across all 100 data sets are almost indistinguishable (Fig. A.1), confirming that the approximation introduced by the tolerance is very minimal. Our ABC-GLM analyses are essentially equivalent to full-likelihood Bayesian analyses, creating a “best-case scenario” for evaluating the marginal likelihood estimates of the ABC-GLM method.

#### Reproducibility

All of the code to replicate our results is freely available at https://github.com/phyletica/abc-glm-marginal-test.
